# EHMTI-0346. Do nurses improve migraine management in primary care?

**DOI:** 10.1186/1129-2377-15-S1-J18

**Published:** 2014-09-18

**Authors:** P van den Berg, B Kollen, P Veenstra, G de Jong

**Affiliations:** 1 Neurology, Isala clinic, Zwolle, Netherlands; 2 Department of General Practice, University Medical Center Groningen, Groningen, Netherlands; 3 Department of Neurology, University Medical Center Groningen, Groningen, Netherlands; 4 Department of Neurology, Isala clinic, Zwolle, Netherlands

## Background

There are indications that a primary health care nurse may improve the treatment of migraine patients.

## Methods

We conducted a non-randomized controlled prospective cohort study in primary care practices. In total 235 patients, diagnosed with migraine with or without aura according to ICHD-2 criteria, between 18 and 65 years of age, were included. Patients with migraine treated only by their general practitioner (control group) were compared to patients managed by a nurse supervised by a general practitioner (intervention group). Primary outcome was the difference in referral rate to a neurologist because of migraine.

## Results

In the intervention group, fewer migraine patients were referred to the neurologist (3.5% vs. 29.8% in the control group, p=0.001). The reduction in mean monthly headache days compared to baseline was apparent in the intervention group at 6 months (6.8 vs. 5.3 in the control group, p=0.006) and 9 months (4.7 vs. 2.1 days in the control group, p=0.006). At 9 months there was no significant change in dichotomized HIT score, compared to baseline (p= 0.068). Change in satisfaction of patients with treatment compared to baseline after 9 months did not differ significantly between the control and intervention group (p=0.070).

## Interpretation

The care administered by a primary care nurse supervised by a general practitioner resulted in less referrals to the neurologist and more headache-free days per month, but no change in HIT score. There was no difference in change from baseline in satisfaction scores between patients of both groups.

No conflict of interest.

